# Barns

**Published:** 1866-04

**Authors:** 


### BARNS.

These should be erected in as dry a locality as possible, where the sun can shine upon them the whole day, and where the ground descends in every direction. Special attention should be paid to the roofing, so that the rain may be turned off rapidly, and that the snow may melt very soon without the possibility of large accumulations.

